# The Biomimetics of Mg^2+^-Concentration-Resolved Microenvironment for Bone and Cartilage Repairing Materials Design

**DOI:** 10.3390/biomimetics7040227

**Published:** 2022-12-05

**Authors:** Zhengqiang Li, Xiaoxue Zheng, Yixing Wang, Tianyi Tao, Zilin Wang, Long Yuan, Bing Han

**Affiliations:** 1 Department of Oral and Maxillofacial Surgery, School and Hospital of Stomatology, Jilin University, Changchun 130021, China; 2 Jilin Provincial Key Laboratory of Tooth Development and Bone Remodeling, Jilin University, Changchun 130021, China; 3 Department of Oral Maxillofacial—Head & Neck Oncology, Shanghai Ninth People’s Hospital Affiliated to Shanghai Jiao Tong University School of Medicine, Shanghai 200011, China; 4 Key Laboratory of Functional Materials Physics and Chemistry of the Ministry of Education, College of Physics, Jilin Normal University, Changchun 130103, China

**Keywords:** magnesium, chondrogenesis, osteogenesis, osteochondral defect, osteochondral unit

## Abstract

With the increase in population aging, the tendency of osteochondral injury will be accelerated, and repairing materials are increasingly needed for the optimization of the regenerative processes in bone and cartilage recovery. The local environment of the injury sites and the deficiency of Mg^2+^ retards the repairing period via inhibiting the progenitor osteogenesis and chondrogenesis cells’ recruitment, proliferation, and differentiation, which results in the sluggish progress in the osteochondral repairing materials design. In this article, we elucidate the Mg^2+^-concentration specified effect on the cell proliferation, osteochondral gene expression, and differentiation of modeling chondrocytes (extracted from New Zealand white rabbit) and osteoblasts (MC3T3-E1). The concentration of Mg^2+^ in the culture medium affects the proliferation, chondrogenesis, and osteogenesis: (i) Appropriate concentrations of Mg^2+^ promote the proliferation of chondrocytes (1.25–10.0 mM) and MC3T3-E1 cells (2.5–30.0 mM); (ii) the optimal concentration of Mg^2+^ that promotes the gene expression of noncalcified cartilage is 15 mM, calcified cartilage 10 mM, and subchondral bone 5 mM, respectively; (iii) overdosed Mg^2+^ leads to the inhibition of cell activity for either chondrocytes (>20 mM) or osteoblasts (>30 mM). The biomimetic elucidation for orchestrating the allocation of gradient concentration of Mg^2+^ in accordance of the physiological condition is crucial for designing the accurate microenvironment in osteochondral injury defects for optimization of bone and cartilage repairing materials in the future.

## 1. Introduction

Bone and cartilage injuries caused by inflammation, trauma, or tumor are one of the major diseases in clinical practice [1,2] and are accelerating with the increasingly aging populations, especially for countries with large populations, such as China. Early cartilage injuries are difficult to be perceived because of the nerveless structure for these tissues. When patients find cartilage damage, the subchondral bone damage is already involved. Thereof, the cartilage damage has become increasingly characterized as a disease of the osteochondral unit rather than simply the cartilage on the articular surface. The swelling, pain, dysfunction, and disability caused by osteochondral defects not only seriously deteriorate people’s quality of life, but also bring heavy economic burden to individual families and society [3,4,5].

Osteochondral defects beyond the critical defects are notorious for being unable to self-heal. Present treatments mainly include physical therapy, drug therapy, and surgery. Physical and drug therapy are only effective for mild patients and can only relieve local symptoms or delay the pathological process and disease progression. These conservative treatments cannot completely restore the osteochondral tissue structure and physiological function [6,7,8]. Current surgical treatments mostly include approaches such as cell transplantation and osteochondral transplantation. However, it still faces a series of clinical problems, such as secondary damage to the donor site, limited donor sources, immune rejection, and disease transmission [9,10,11]. Meanwhile, the repaired tissue is mainly fibrocartilage, which has a poor capacity to bear loading and is easy to degenerate [12]. These traditional treatments are hard to reconstruct the hierarchical structures and restore the functions of the osteochondral unit.

Tissue engineering has brought new opportunities for the repair of osteochondral defects. As the central component in osteochondral tissue engineering, a suitable microenvironment plays an important role in providing physical, mechanical, and biochemical signals guiding the seed cells’ migration, proliferation, differentiation, and secretion of extracellular matrix (ECM) to repair the osteochondral unit [13]. Many attempts have tried to reconstruct the microenvironment of the osteochondral unit. Hierarchical structures constructed from double-layer, three-layer, or gradient scaffolds aim to mimic the structural microenvironment, while composite scaffolds constructed from organic and inorganic materials are used to mimic the compositional microenvironment [14]. However, the repaired tissue is usually fibrocartilage, and it cannot integrate well with the subchondral bone, which manifests as the loss or disorder of tidemark and calcified cartilage. This may be because the scaffold mainly provides physical and mechanical signals via the microstructure and surface topography. Compared with the physical and mechanical signals, the biochemical signals provided by endogenous or exogenous cytokines (e.g., growth factors and immunomodulatory factors), hormones, and microelements are the crucial factors regulating the biological behavior of cells in the microenvironment [13]. Various growth factors such as BMP-2 and TGF-β are used to obtain better osteochondral repair effects. However, they have recently become increasingly objectionable by the FDA to approve the use of such growth factors due to safety concerns, as they can lead to ectopic osteogenesis or unwanted bone formation [15,16,17]. TGF-β can also interfere with the homeostasis of subchondral bone and even lead to the occurrence of osteoarthritis [18]. Other growth factors such as fibroblast growth factor, insulin-like growth factor, and vascular endothelial growth factor may cause cartilage calcification. The FDA’s review of such biologics has become more stringent, which makes their commercial viability more difficult and expensive [19]. In osteoarthritis, in addition to local osteochondral defects, the local aseptic inflammatory microenvironment further hinders the repair of the osteochondral unit [20].

Magnesium (Mg) is an essential element of the human body that plays important role in various life activities. Various enzymes in the body can play their physiological metabolism under the action of Mg, such as Na^+^/K^+^—ATPase, hexokinase, creatine kinase, protein kinase, cyclase, etc. [21]. Mg acts a pivotal part in nerve and neuromuscular conduction, cardiac excitation, muscle contraction, vasomotion, blood pressure regulation, glucose, and insulin metabolism. Mg also plays a significant role in cartilage and bone development, metabolism, and regeneration. About 50–60% of Mg is present in bones, and Mg accounts for 0.5–1% of bone mineral content. It can not only affect the metabolism of bone minerals and matrix by regulating bone metabolism-related hormones, growth factors, and signaling pathway-related factors, but also directly affect the bone tissue itself [22]. At the same time, the study also found that Mg has a chondroprotective effect. Mg can reduce the loss of type II collagen and glycosaminoglycan in osteoarthritis, and can inhibit the expression of interleukin-1β (IL-1β), Tumor Necrosis Factor-α (TNF-α), and matrix metalloproteinase 13 (MMP13) in osteoarthritis [23]. Mg deficiency caused a marked reduction in the number and size of chondrocytes in the proximal femur, and the femur growth plate lacked an orderly cartilage column arrangement and was significantly reduced in width [24]. Reduced levels of proteoglycan and SRY-Box Transcription Factor 9 (SOX9) in both articular cartilage and growth plate extracellular matrix in rats subjected to Mg dietary restriction for 6 months [20,25].

Therefore, considering the important roles of Mg in cartilage and bone development, we can construct an Mg-enriched microenvironment with a concentration gradient. Different concentrations of magnesium ion (Mg^2+^) regulate the biological behaviors of the cells (chondrocytes, hypertrophic chondrocytes, and osteoblasts) in different layers (articular cartilage (90% in volume), calcified cartilage (5%), and subchondral bone (5%)) in the osteochondral unit to better repair osteochondral defects. However, the precise regulation of Mg^2+^ on the biological behavior of osteochondral tissue engineering seed cells has not been systematically studied. So, in this experiment, we aimed to systematically investigate the effect of different concentrations of Mg^2+^ on the proliferation, chondrogenesis, and osteogenesis of chondrocytes and pre-osteoblasts. We also investigated the anti-inflammatory effects of Mg^2+^ to use it for osteochondral defect regeneration in osteoarthritis.

## 2. Results

### 2.1. Effects of Mg^2+^ on Chondrocytes

#### 2.1.1. Cell Proliferation

**Figure 1:**
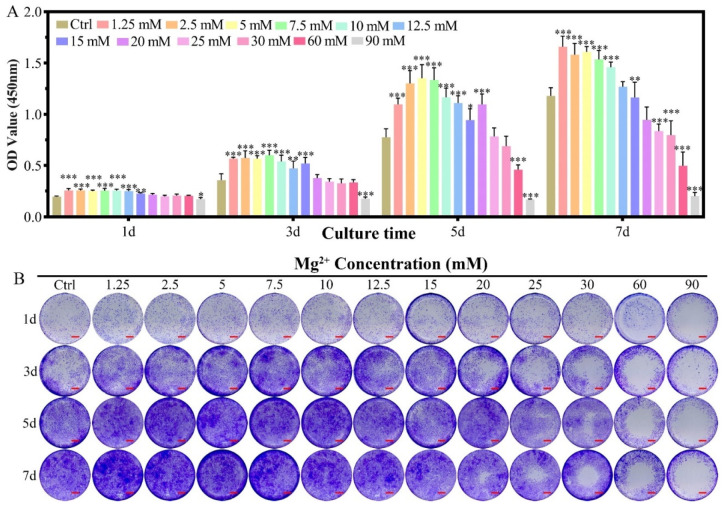
The proliferation of chondrocytes cultured in varied Mg^2+^ concentrations from 0.7 mM (Ctrl group) up to 90.0 mM. (**A**) the CCK-8 quantitative method of the cell proliferation, all data were presented as mean ± SD, * *p* < 0.05, ** *p* < 0.01, and *** *p* < 0.001; (**B**) digital images of the crystal violet stained chondrocytes cultured in different Mg^2+^ containing DMEM/F12 medium, scale bar = 1 mm.

The effect of Mg^2+^ concentration ([Mg^2+^]) on the proliferation of chondrocytes was assessed by the CCK-8 method in reference to that cultured in DMEM/F12 without any more Mg^2+^ addition (Figure 1A). For chondrocytes of New Zealand white rabbit, providing certain amounts of Mg^2+^ supplementation promoted cell proliferation (1.25–15 mM) from the 1st to the 5th day, while the promotion effect was getting reduced in higher concentrations (15–20 mM), especially for long culture duration. The [Mg^2+^] on the proliferation of chondrocytes showed three different concentration-dependent results: (i) promoted proliferation of chondrocytes in the range of 1.25 ≤ [Mg^2+^] ≤ 10.0 mM till the 7th day; (ii) the promotion effect gradually reduced by the 7th day in the range of 12.5 ≤ [Mg^2+^] ≤ 20.0 mM; (iii) have no promotion effect all the time for [Mg^2+^] ≥ 25.0 mM. The highest proliferation result was found at 7.5 mM on the third day, 5.0 mM on the fifth day, and 1.25 mM on the seventh day. These results demonstrated that the long-lasting promotion effect on chondrocyte proliferation should avoid high [Mg^2+^] in the initiation of the culture. The optimum [Mg^2+^] range is 1.25–15.0 mM.

In the crystal violet staining results, the same tendency could be observed as that measured from the CCK-8 method. The increased purple color for the concentration range of 1.25–10.0 mM demonstrated the proliferation effect of Mg^2+^ at this range (Figure 1B). With increasing the concentration of Mg^2+^, the live chondrocytes increased with low concentration and dead with increasing the Mg^2+^ concentration, especially for the concentration of 25.0 mM. The live/dead staining for the concentration of Mg^2+^ over 30.0 mM was excluded due to the high dead ratio according to CCK-8 results (Figure S2A,C). Morphology of the chondrocytes kept unchanged for the Mg^2+^ concentration up to 25.0 mM according to FITC-phalloidin and DAPI staining results (Figure S1A and Figure S2B).

#### 2.1.2. Chondrogenic-Related Gene Expressions

**Figure 2:**
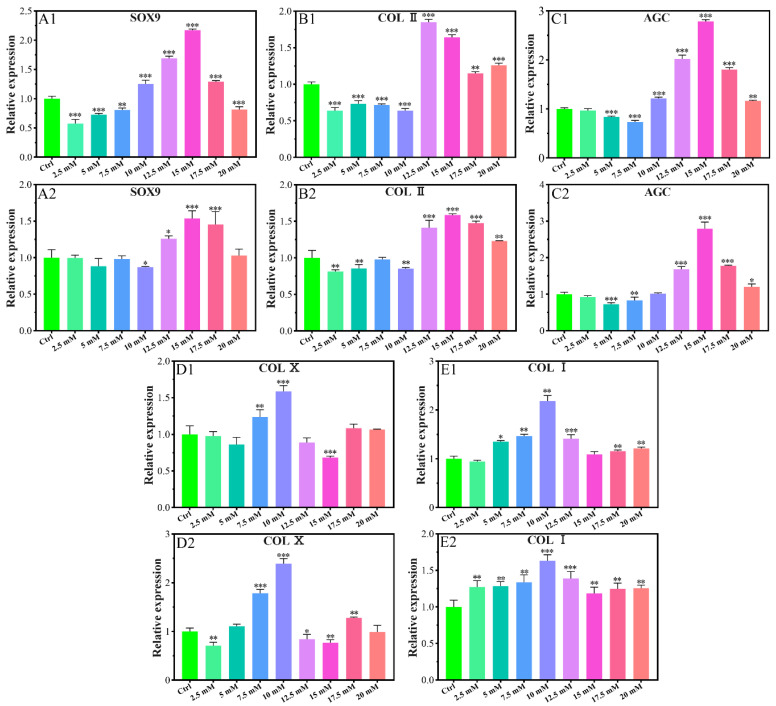
Effects of gene expression of chondrocytes cultured in different Mg^2+^ concentrations on the 7th day (**A1**,**B1**,**C1**,**D1**,**E1**) and the 14th day (**A2**,**B2**,**C2**,**D2**,**E2**). All data are presented as fold change after normalization to GAPDH, and fold changes are shown as mean ± SD, * *p* < 0.05, ** *p* < 0.01, and *** *p* < 0.001.

The expression of SOX9 gene was promoted for the concentration of 10–17.5 mM of Mg^2+^ on the 7th day (Figure 2A1) and 12.5–17.5 mM of Mg^2+^ on the 14th day (Figure 2A2). All of the other Mg^2+^ concentrations did not promote the expression of the SOX9 gene.

The expression of the collagen type II (COL II) gene was inhibited in the concentration range of 2.5–10.0 mM on the 7th day (Figure 2B1), and that of 2.5–10.0 mM on the 14th day with a slight exception concentration of 7.5 mM (Figure 2B2). The gene expression promotion concentration range was 12.5–20.0 mM on the 7th day with the highest value for the concentration of 12.5 mM, which kept up to the 14th day with the highest expression for that of 15.0 mM.

The expression of the aggrecan (AGC) gene was also promoted in a similar range as that of SOX9, i.e., 10.0–20.0 mM on the 7th day (Figure 2C1), and 12.5–20.0 mM on the 14th day with the highest value for the concentration of 15.0 mM of Mg^2+^ (Figure 2C2).

The gene expression of collagen type X (COL X) was promoted only for the concentration of 7.5 mM and 10.0 mM on the 7th day (Figure 2D1) and the fourteenth day. 17.5 mM concentration of Mg also had a promoting effect on the 14th day (Figure 2D2). For all of the other concentrations of Mg^2+^ at any time, the expression of COL X was inhibited or not changed according to the data.

The expression of the collagen type I (COL I) gene was promoted in the concentration of Mg^2+^ of 5.0–12.5 mM and 17.5–20.0 mM on the 7th day (Figure 2E1), and that range of 2.5–20.0 mM on the 14th day (Figure 2E2). The most expression result was 10 mM of Mg^2+^ on either the 7th day or the 14th day.

#### 2.1.3. Chondrogenic-Related Protein Expressions

**Figure 3:**
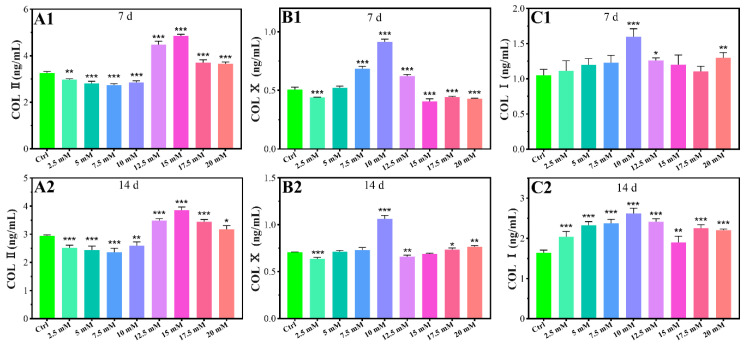
Effects of protein contents of chondrocytes cultured in different Mg^2+^ concentrations on the 7th day (**A1**,**B1**,**C1**) and the 14th day (**A2,B2**,**C2**), all data were presented as mean ± SD, * *p* < 0.05, ** *p* < 0.01, and *** *p* < 0.001.

The protein levels of COL Ⅱ, COL Ⅹ, and COL Ⅰ were studied using chondrocytes cultured for 7 days and 14 days respectively. The levels of COL Ⅱ protein significantly increased in high concentrations of Mg^2+^ (≥12.5 mM) compared with the control group for 7 days and 14 days, while low concentrations of Mg^2+^ (≤10 mM) inhibited the levels of COL Ⅱ protein (Figure 3A1,A2). At a concentration of 15 mM, Mg^2+^ significantly increased COL Ⅱ protein contents 1.49- and 1.31-fold, for 7 days and 14 days respectively, when compared to the control group (Figure 3A1,A2). As shown in Figure 3B1,B2, the levels of COL Ⅹ protein significantly increased with 7.5 mM-12.5 mM and 10 mM Mg^2+^ treatment for 7 days and 14 days respectively. At a concentration of 10 mM, Mg^2+^ significantly increased COL Ⅹ protein contents 1.80- and 1.51-fold for 7 days and 14 days compared to the control group. Treatment with 10 mM, 12.5 mM, and 20 mM Mg^2+^ for 7 days significantly increased the COL I protein levels for 7 days compared to the control, while treatment with Mg^2+^ significantly increased the COL I protein levels for all concentrations for 14 days (Figure 3C1,C2).

#### 2.1.4. Inflammatory-Related Gene Expressions

**Figure 4:**
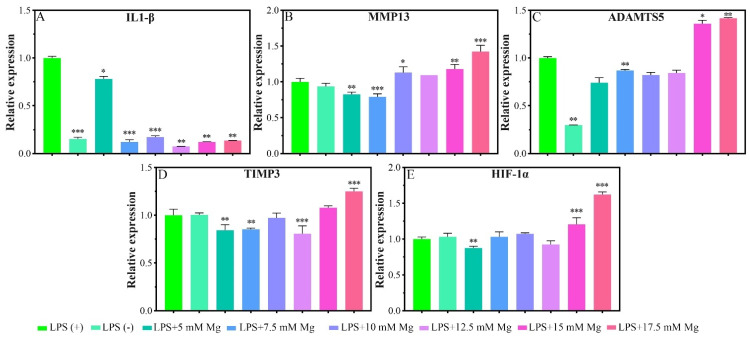
Inflammatory-related gene expressions of chondrocytes that cultured in different concentrations of Mg^2+^. All data are presented as fold change after normalization to GAPDH, and fold changes are shown as mean ± SD, * *p* < 0.05, ** *p* < 0.01, and *** *p* < 0.001.

Given the pathological characteristics of chondrocytes in osteoarthritis, we focused on the effects of Mg^2+^ concentration on IL-1β, MMP13, a disintegrin and metalloproteinase with thrombospondin motifs 5 (ADAMTS5), metalloproteinase inhibitor 3 (TIMP3), and hypoxia-inducible factor 1-alpha (HIF-1α) in inflammatory chondrocytes. Lipopolysaccharide (LPS) could serve as a reliable proxy for infection-induced inflammation via Toll-like receptors (TLR) as a prospective mode to induce the inflammation [26]. In the inflammatory model, the LPS (+) group showed a significant expression of IL-1β and ADAMTS5 genes in reference to the LPS (−) group, which indicated the model was successfully built in this experiment (Figure 4A). Cells cultured in different Mg^2+^ concentration conditions showed a significant inhibiting effect for the expression of the IL-1β gene in comparison with that of the LPS (+) group (Figure 3A). The expression of MMP13 was inhibited for the concentration range of 5.0–7.5 mM of Mg^2+^ and promoted for the range of 15.0–17.5 mM (Figure 4B). The expression of the ADAMTS5 gene was inhibited for the concentration of 7.5 mM and promoted for the range of 15.0–17.5 mM (Figure 4C). The inhibition range for TIMP3 expression was 5.0 mM, 7.5 mM, and 12.5 mM, which turned to slight promotion for the range of 17.5 mM (Figure 4D). The inhibition effect for the HIF-1α gene was not obvious as the previous genes, which showed slight inhibition for the concentration of 5.0 mM, and also promoted for the range of 15.0–17.5 mM of Mg^2+^ (Figure 4E).

### 2.2. Effects of Mg^2+^ on MC3T3-E1 Cells

#### 2.2.1. Cell Proliferation

**Figure 5:**
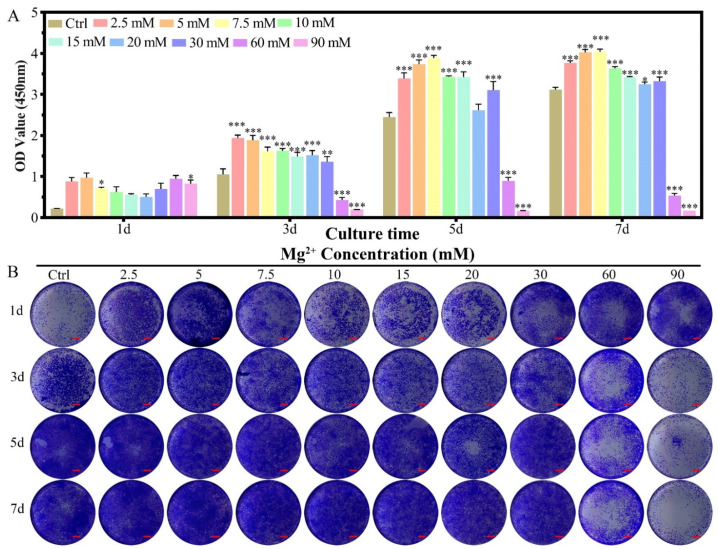
The proliferation of MC3T3-E1 cells cultured in varied Mg^2+^ concentrations from 0.8 mM (Ctrl group) up to 90.0 mM. (**A**) the CCK-8 quantitative method of the cell proliferation, all data were presented as mean ± SD, * *p* < 0.05, ** *p* < 0.01, and *** *p* < 0.001; (**B**) digital graphs of the crystal violet stained MC3T3-E1 cells cultured in different Mg^2+^ containing culture medium, scale bar = 1 mm.

The proliferation of MC3T3-E1 cells showed an Mg^2+^ concentration-dependent phenomenon for the culture in seven days (Figure 5A). The introduction of Mg^2+^ in the culture medium for MC3T3-E1 cells promoted the proliferation on the first day for all of the concentration groups, which kept the trend up to the 7th day for the concentration range of 2.5–30 mM. An inhibition effect was found for the concentration range of 60.0–90.0 mM from the 3rd day to the seventh day. In the crystal violet staining results, the same tendency could be observed as that measured from the CCK-8 method (Figure 5B). In the live/dead cell staining results, the dead cell ratio increased slightly with increasing Mg^2+^ concentration, which showed a significant dead amount for the concentration over 30 mM (Figure S3A,C). The morphology of the cells was nearly unchanged for the full range of the Mg^2+^ concentration (Figure S1B and Figure S3B).

#### 2.2.2. Osteogenic-Related Gene Expressions

**Figure 6:**
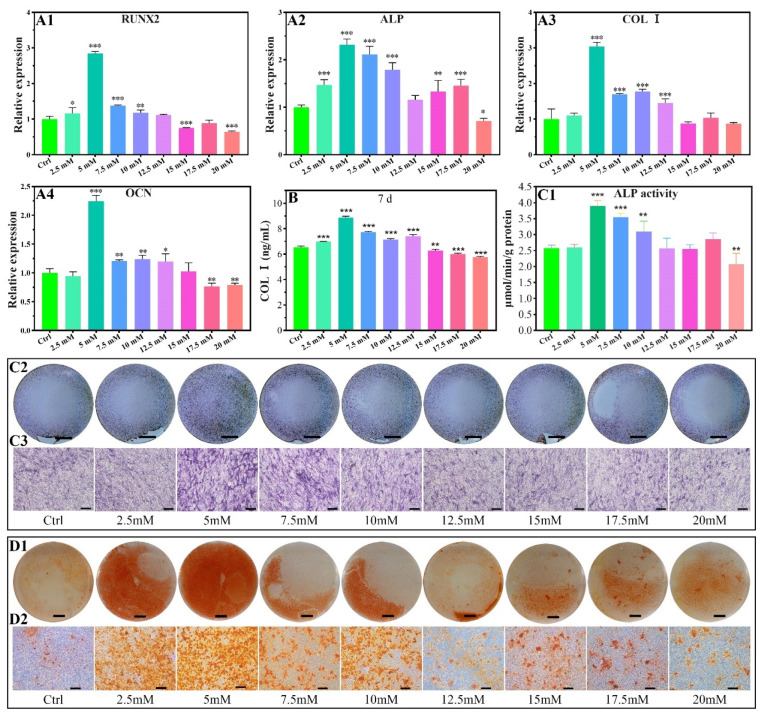
Osteogenic-related gene expressions of MC3T3-E1 cells cultured in different concentrations of Mg^2+^ (**A1**–**A4**), all data are presented as fold change after normalization to GAPDH, and fold changes are shown as mean ± SD, *p < 0.05, **p < 0.01, and ***p < 0.001. COL I contents of MC3T3-E1 cells after being cultured in varied Mg^2+^ concentrations on the 7d by ELISA (**B**). ALP activity of MC3T3-E1 cells after being cultured in varied Mg^2+^ concentrations on the 7d by quantitative ALP activity assay (**C1**) and ALP activity staining assay (**C2**,**C3**). Effect of Mg^2+^ concentrations on the formation of calcium nodules (**D1**,**D2**). The scale bars in C2, C3, D1, and D2 are 5 mm, 200 μm, 5 mm, and 200 μm, respectively.

The expression promotion range of the Runt-related transcription factor 2 (RUNX2) gene was 2.5–10 mM of Mg^2+^ on the seventh day (Figure 6A1). The most promotion results occur for the concentration of 5.0 mM. No significant promotion effect for the expression of the RUNX2 gene in the concentrations of 12.5 and 17.5 mM, and that changed as an inhibition for the concentrations of 15.0*–*20 mM.

The gene of alkaline phosphatase (ALP) showed a slight promotion of the expression in the Mg^2+^ concentration range of 2.5–10.0 mM and 15–17.5 mM, with other concentrations showing an inhibition or no promoting effect (Figure 6A2).

The expression of collagen type I (COL I) in MC3T3-E1 cells was promoted for the Mg^2+^ concentration range of 5–12.5 mM, which had no promoting effect in the range of 15.0–20.0 mM. The highest expression occurred for the 5 mM of Mg^2+^ (Figure 6A3).

The expression of osteocalcin (OCN) was significantly promoted for the concentration of 5*–*12.5 mM, and other concentrations showed no obvious promoting effect or are inhibited for the 2.5 mM, 15.0–20 mM (Figure 6A4).

#### 2.2.3. COL I Contents

The protein levels of COL Ⅰ were studied using MC3T3-E1 cells cultured for 7 days. As shown in Figure 6B, the levels of COL Ⅰ protein significantly increased with 2.5 mM-12.5 mM Mg^2+^ treatment, while the levels of COL Ⅰ protein were inhibited by 15–20 mM Mg^2+^. At a concentration of 5 mM, Mg^2+^ significantly increased COL Ⅰ protein contents 1.36-fold compared to the control group (Figure 6B).

#### 2.2.4. ALP Activity

ALP activity by quantitative analysis and staining showed a promotion effect for the concentration range of 5–10 mM. An inhibition effect was found for the concentration up to 20 mM (Figure 6C1–C3).

#### 2.2.5. Calcium Nodules Formation

The calcium nodules increased for the Mg^2+^ concentration range of 2.5–10.0 mM in reference to the control group (Figure 6D1,D2), indicating the introduction of Mg^2+^ promotes calcium nodules formation.

## 3. Discussion

**Figure 7:**
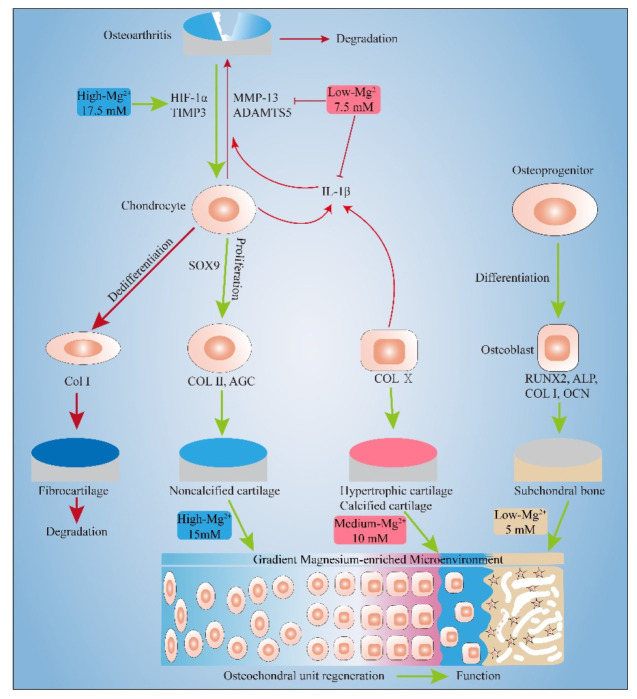
Gradient Mg^2+^-enriched Microenvironment promotes the formation of Noncalcified cartilage, hypertrophic cartilage and subchondral bone. Appropriate concentrations of Mg^2+^ also have an anti-inflammatory effect and pro-anabolism effect.

In this experiment, the effects of different concentrations of Mg^2+^ on the chondrocytes and MC3T3-E1 cells were systematically studied for the first time. It was found that certain concentrations of Mg^2+^ can promote the proliferation of chondrocytes (≤10 mM) and MC3T3-E1 cells (≤30 mM). Appropriate concentrations of Mg^2+^ can also have an anti-inflammatory effect (7.5 mM) and pro-anabolism effect (17.5 mM) on Inflammatory chondrocyte. Moreover, gradient concentrations of Mg^2+^ can promote the expression of the markers of noncalcified cartilage (15 mM), calcified cartilage (10 mM), and subchondral bone (5 mM), which is beneficial to the regeneration of osteochondral units (Figure 7).

### 3.1. Pro-Proliferation Effect of Mg^2+^ on Chondrocytes and MC3T3-E1 and Its Possible Mechanism

The concentration of Mg^2+^ has been found to directly affect cell proliferation either in chondrocytes or MC3T3-E1 cells. For chondrocytes of New Zealand white rabbit, providing certain amounts of Mg^2+^ supplementation promotes cell proliferation (1.25–15 mM) from the 1st to the 5th day, while the promotion effect was getting reduced in higher concentrations, especially for long culture duration (Figure 1). These results agree with that of the concentration-dependent proliferation effect of MgSO_4_ (5–10 mM) on the human articular chondrocytes [27]. The regulatory effect of Mg^2+^ concentration on chondrocyte proliferation has been reported in porcine and human chondrocyte cultures [27,28]. However, there is still some controversy about the specific concentration range of chondrocyte proliferation. Especially for the New Zealand white rabbit as a model animal for classical osteochondral repair research, there is no relevant report. We systematically adjusted the Mg^2+^ concentration in the culture environment of New Zealand white rabbit chondrocytes and found that 1.25–10 mM Mg^2+^ can promote the proliferation of chondrocytes.

Then we investigated the relationship between MC3T3-E1 cell proliferation and Mg^2+^ concentration. The results found that 2.5–30 mM Mg^2+^ can promote the proliferation of MC3T3-E1 cells, which could tolerate slightly higher concentrations of Mg^2+^ than chondrocytes (30 mM vs. 10 mM). When the Mg^2+^ concentration is greater than 60mM, MC3T3- E1 showed obvious cytotoxicity (Figure 5). Although the stimulating role of magnesium phosphate ceramic has been found in increasing the proliferation rate of MC3T3-E1, there is no relevant research on the effect of Mg^2+^ concentration on the proliferation of MC3T3-E1 cells in the literature [29].

The synthesis of proteins is highly dependent on the concentration of intracellular Mg^2+^ due to the Mg^2+^ as a co-enzyme for many important cell cycle processes. Increasing the concentration of Mg^2+^ has been convicted that promoting DNA and protein synthesis previously [30]. The regulation mechanism of the pro-proliferation effect of Mg^2+^ on the two kinds of cells is the same. The effects of Mg^2+^ on the cell proliferation and differentiation cycle have been summarized in the literature: (i) Moderate concentration of Mg^2+^ promotes the mitosis of the cells by activating the Mg^2+^ transporter at the membrane and stimulating the synthesis of DNA and proteins by providing more Mg-ATP co-enzymes [31]; (ii) Deficiency of Mg^2+^ concentration leads to the upregulation of the cyclin inhibitor p27Kip1, which in turn inhibits the activity of cyclin E-dependent kinases, resulting in blocked DNA and protein synthesis, and cell growth arrest [24,32]; The inhibition effect of high concentration of Mg^2+^ in chondrocyte proliferation could be ascribed to the locally distorting of the double helix of DNA due to the covalent binding with Mg^2+^ in the nucleus [33]. (iii) excessive Mg^2+^ concentration leads to the increased osmotic pressure of the culture medium, and Mg^2+^ has an antagonistic effect on calcium-related signaling proteins as cell membrane receptors and second messengers, thereby triggering cell membrane rupture and cell death [34]. Although the regulation of cell proliferation by Mg has a certain common law, for different cells, the range of its pro-proliferation concentration is still quite different, which may be related to the difference in the number of Mg^2+^ transporters on cell membrane between different species.

As we know, chondrocyte hypertrophy is marked by a 10-fold increase in cell volume [35]. To our surprise, although the gene and protein levels of hypertrophic chondrocyte marker COL X elevated when the culture medium contain 7.5–10 mM Mg^2+^, we did not observe significant morphology changes of chondrocytes cultured in the culture plates. This may be due to the high cell seeding density which result in insignificant change in cell morphology, or it may be related to the two-dimensional culture environments. We will further optimize the cell seeding density and change the cell culture environment to observe the effect of Mg^2+^ on the change of chondrocyte morphology.

### 3.2. Effect of Mg^2+^ Concentration on Chondrogenesis of Chondrocytes and Its Mechanism

Given the histological and physiopathological characteristics of articular cartilage, we focused on the effect of Mg^2+^ concentration on gene expressions of SOX9, COL II, AGC, COL X, and COL I in chondrocytes. The subcultured chondrocytes exhibit certain differentiation plasticity. They can change their developmental fate such as dedifferentiation and transdifferentiation under different regulatory mechanisms. SOX9, COL II, and AGC are markers of chondrogenic differentiation, while COL X and COL I are markers of chondrocytes hypertrophy and dedifferentiation, respectively.

As shown in Figure 2A–C, in the concentration range of 12.5–17.5mM of Mg^2+^, SOX9 gene expression can be promoted, while COL II and AGC genes are expressed higher compared to the control group in Mg^2+^ concentrations in the range of 12.5–20 mM. 15 mM Mg^2+^ has the best effect on promoting the expression of the three genes. ELISA results showed that 12.5–20 mM of Mg^2+^ promoted the protein levels of COL II, and 15 mM of Mg^2+^ had the best promotion effect (Figure 3A1,A2). SOX9, COL II, and AGC have a similar Mg^2+^ concentration-regulated expression range, which is because SOX9 is a regulatory transcription factor in the process of chondrogenesis, and can positively regulate the expression of COL II and Aggrecan by binding to the specific enhancers of COL II and AGC [36,37]. COL II is the most important protein in hyaline cartilage, which forms a three-dimensional network structure in the extracellular matrix with hydrophilic acidic aggrecan loosely bonded [38].

As shown in Figure 2D1,D2, when the concentration of magnesium is 2.5 mM, 12.5 mM and 15 mM, the gene expression of COL X can be inhibited, indicating that magnesium in this range inhibits chondrocyte hypertrophy; At 7.5–10 mM, the gene expression of COL X was promoted, indicating a pro-hypertrophy effect of magnesium in this range. Protein quantification results by ELISA showed that 10 mM Mg^2+^ promote the COL X protein levels (Figure 3B1,B2). Studies have found that Mg^2+^ can inhibit chondrocyte hypertrophy and matrix calcification by regulating the MAPK/ERK phosphorylation signaling pathway and autophagy [39], which is closely related to the inhibition of COL X gene expression. In this study, we found that whether Mg^2+^ promotes or inhibits COL X expression is related to the dose of Mg^2+^. 10 mM Mg^2+^ had the best promotion effect on the COL X gene and protein expression levels. COL X is the main marker of hypertrophic cartilage and calcified cartilage. It plays several roles including stabilizing the remodelling ECM [40], regulating the distribution of collagens and proteoglycans throughout the ECM [41], and regulating the zonal distribution of matrix vesicles [42]. Runx2 can regulate the expression of COL X in pre-hypertrophic and hypertrophic chondrocytes, which can bind and release calcium in a dose-dependent manner, thereby participating in endochondral ossification [43], which is beneficial to the bone formation during bone development. In osteoarthritis, the amount of hypertrophic cartilage is also increased with an up-regulation of COL X. Therefore, the regulation of Mg^2+^ on chondrocyte COL X is bidirectional, and the change of Mg^2+^ concentration can be adjusted according to the purpose of treatment. We can design scaffolds that contain appropriate concentrations of Mg^2+^ by 3D printing or hydrogels to repair bone defect by promoting endochondral osteogenesis or promote cartilage regeneration by inhibiting chondrocyte hypertrophy. For patients with osteoarthritis, the local delivery of appropriate concentrations of Mg^2+^ by local injection of nanocarrier to inhibit the up-regulation of COL X may reduce the progression of osteoarthritis.

The expressions of the COL I gene and protein were elevated when the Mg^2+^ concentration is higher than 0.7 mM (Figure 2E1,E2 and Figure 3E1,E2), which indicates that the high Mg^2+^ microenvironment can cause the dedifferentiation of chondrocytes. One of the disadvantages of the in vitro culture of chondrocytes is the potential of dedifferentiation, especially for the cells cultured in a two-dimensional environment in vitro or in more generations of propagation for a long time. The morphology of the dedifferentiated chondrocytes changes from triangle to the elongated spindle, with the main collagen changing from type-II to type-I [44]. The occurrence of COL I, as the main collagen protein of fibrous cartilage, indicates poor quality of the cartilage, which is prone to degenerate and unable to fulfill its function [45,46]. However, the main type of collagen in fibrocartilage such as articular disc and condyle is COL I. Magnesium may promote the regeneration of fibrocartilage by promoting the production of COL I, so it may also be used for condylar cartilage and articular disc regeneration [47]. In our case, the supplement of Mg^2+^ does not inhibit the dedifferentiation of chondrocytes of New Zealand white rabbit. But as we know, chondrocytes are prone to dedifferentiation when cultured in two-dimensional environments, while they can return to the phenotype of normal chondrocytes in three-dimensional environments. The dedifferentiation of chondrocytes characterized by up-regulation of COL I, which may be related to the two-dimensional culture environment in this study. We will use three-dimensional culture environments such as pellet culture and hydrogel culture to study the effect of Mg^2+^ on chondrocytes dedifferentiation in further study.

Although the repair of osteochondral defects is accomplished by bone marrow mesenchymal stem cells, it is also accompanied by the formation of fibrocartilage, which reduces the quality of the neo-cartilage. In the clinical trials for patients with cartilage defects, the use of chondrocyte transplantation has achieved good results. If Mg^2+^ can promote the migration of surrounding chondrocytes to the defect area and continuously secrete cartilage matrix to repair cartilage defects, then we do not need to expand chondrocytes in vitro, but directly implant the Mg^2+^-based biomaterials rely on endogenous chondrocytes to repair cartilage layer defects. This may be a method that can be considered in the future.

### 3.3. Effect of Mg^2+^ Concentration on Inflammation Related Gene Expressions and Its Mechanism

As shown in Figure 4, the inhibitory concentration ranges of Mg^2+^ on IL-1β, MMP13, and ADAMTS5 were 5–17.5 mM, 5–7.5 mM, and 7.5 mM, respectively. The inhibitory concentration of Mg^2+^ on the above genes is lower than that of promoting effect of ECM synthesis of chondrocytes.

IL-1β is the main inflammatory factor related to the occurrence and development of osteoarthritis. It can promote the synthesis of MMP13 and ADAMTS5, thereby degrading collagen type II fibrils and proteoglycan [48]. Studies have shown that Mg^2+^ may affect osteoarthritis by inhibiting the expression of IL-1β, TNF-α, MMP13, and ADAMTS5 genes in macrophages, synoviocytes, and chondrocytes [23,24,39]. Mg^2+^ can inhibit the expression of IL-1β and TNF-α in the synovial tissue of New Zealand rabbits with OA by inhibiting the calcium ion channel TRPV5 and activating cartilage autophagy [49]. The inhibitory effect of Mg^2+^ on IL-1β, MMP13, and ADAMTS5 indicates that it has a certain potential in the treatment of osteoarthritis and 5–7.5 mM Mg^2+^ was most effective in inhibiting cartilage catabolism.

This study also found that low concentrations of Mg^2+^ (5 mM, 7.5 mM, 12.5 mM) could inhibit the expression of TIPM3, while high concentrations of Mg^2+^ (17.5 mM) could promote the expression of TIPM3. Zan et al. found that 20 mM Mg^2+^ chloride can promote the synthesis of TIPM3 [50], which is almost the same as the concentration that promotes the expression of TIPM3 in this paper. TIPM3 is a major proteoglycanase inhibitor. High concentrations of Mg^2+^ promote the synthesis of TIPM3 to further suppresses osteoarthritis by inhibiting the expression of ADAMTS4, ADAMTS5, and MMP-13 [51,52].

Given the physiological characteristics of the hypoxia environment in cartilage, we studied the effect of [Mg^2+^] on the expression of HIF-1α in inflammatory chondrocytes and found that 5 mM of [Mg^2+^] had a certain inhibitory effect on the expression of HIF-1α, while 15 and 17.5 mM of [Mg^2+^] could promote the expression of HIF-1α (Figure 4E). The effects of [Mg^2+^] on HIF-1α and TIPM3 tend to be almost the same, probably because HIF-1α can act on the promoter of TIPM3, thereby increasing its transcription and protein expression [53]. Studies have shown that a hypoxic environment plays an important role in promoting cartilage repair and regeneration. For example, co-immunoprecipitation confirmed that HIF-1α and SOX9 combined with each other to promote cartilage formation. The activation of SOX9 can be inhibited after knocking out the HIF-1α promoter site. The expression of SOX9 was increased twofold under hypoxic conditions relative to normoxic conditions [54,55]. HIF-1α also can maintain chondrocyte and cartilage homeostasis by regulating chondrocyte autophagy and apoptosis [56]. Therefore, the promotion of HIF-1α expression by Mg^2+^ is beneficial to the synthesis of cartilage matrix and the maintenance of cartilage homeostasis. Another study found that HIF-1α inhibits the expression of MMP13 by blocking the interaction between transcription factor 4 and β-catenin, while the reduction of HIF-1α can promote the expression of MMP13 and increase cartilage damage [57]. In this study, the Mg^2+^ concentration that promotes HIF-1α expression also promotes MMP13 expression, although the mechanism remains to be further studied, this problem can be solved by trying to combine drugs such as zinc-binding and non-zinc-binding selective MMP-13 inhibitors that can inhibit MMP-13 expression in the treatment of osteoarthritis.

In the case of osteochondral defects in arthritis, the inflammatory factors that degrade the cartilage matrix should be inhibited first, and then promote the synthesis of cartilage matrix. Since the negatively charged glycosaminoglycans in the cartilage matrix can adsorb a large amount of positively charged Mg^2+^, Mg^2+^ enriched in the cartilage matrix gradually increase with the degradation of the Mg-doped scaffold. [Mg^2+^] changes from low to high, which is in line with the sequence of low concentrations inhibiting inflammation firstly, followed by high concentrations promoting cartilage matrix synthesis. We have fabricated scaffolds that contain appropriate concentrations of Mg^2+^ by 3D printing or hydrogels. The gradient distribution of Mg^2+^ can be achieved by two-phase, three-phase, or gradient scaffold.

### 3.4. Effect of Mg^2+^ Concentration on Osteogenesis of MC3T3-E1 Cells and Its Mechanism

Aiming at the histological and physiological characteristics of bone, we focused on the effects of Mg^2+^ concentration on the gene expressions of RUNX2, ALP, COL I, and OCN in MC3T3-E1 cells. As shown in Figure 6A1–A4, RUNX2 gene expression was promoted when the Mg^2+^ concentration was less than 10 mM, while the ALP, Collagen I, and OCN genes were up-regulated in the range of Mg^2+^ concentration in 2.5–10 mM, 5–12.5 mM, and 5–12.5 mM, respectively. The regulation of these gene expressions has similar Mg^2+^ concentration ranges, which is because RUNX2 can bind to osteoblast-specific cis-acting elements to regulate the transcription and translation of ALP, Collagen I, and OCN, thereby promoting cell growth, osteogenesis, and accelerated extracellular matrix deposition [58].

At the same time, the protein levels of COL I and ALP were measured. COL I is the most abundant protein in bone matrix, while ALP plays an important role in regulating bone mineralization. ELISA results showed that 2.5–12.5 mM Mg^2+^ promoted the expression of COL I protein, while 15–20 mM Mg^2+^ inhibited the expression of COL I protein (Figure 6B). Through the quantitative and qualitative experiments of ALP activity, we found that 5–10 mM Mg^2+^ can promote the synthesis on ALP protein (Figure 6C1–C3). The protein quantification results were consistent with the PCR results. Under the activation of Mg^2+^, ALP can hydrolyze phosphate monoesters and other substrates to release phosphate. The accumulation of local phosphate ions results in excess soluble calcium phosphate products and calcium phosphate precipitation. Calcium salts form calcification centres along COL I fibres and hydroxyapatite are gradually formed to form osteoid under the control of non-collagen such as OCN.

The number of calcium nodules formed by 2.5–10 mM Mg^2+^ was significantly more than other groups, while higher concentrations of Mg^2+^ inhibited calcium nodules formation (Figure 6D1,D2), probably because Mg^2+^ is an antagonist of calcium. High concentrations of extracellular Mg^2+^ can reduce the calcium content in mitochondria, inhibit the formation of matrix vesicles and autophagosomes, and ultimately inhibit the formation of bone matrix. Compared with the concentration range of Mg^2+^ promoting cartilage extracellular matrix synthesis. The concentration range of Mg^2+^ promoting bone matrix synthesis in MC3T3-E1 cells is relatively lower, which may be due to the difference in the number of Mg^2+^ transporters and receptors on different cell membranes.

In this study, we found that 5 mM Mg^2+^ had the best effect on promoting osteogenesis according to osteogenesis-related gene expression, COL I protein contents, ALP activity, and calcium nodule formation.

In the future, we can develop multi-layered scaffolds or gradient scaffolds for the regeneration of articular osteochondral defects. Each layer of the scaffolds corresponds to the hyaline chondrocyte layer, the hypertrophic chondrocyte layer, and the subchondral bone layer, respectively. In each layer of the scaffold, different concentrations of Mg^2+^ are loaded and the release of specific concentrations of Mg^2+^ is precisely regulated. The different effects of different concentrations of Mg^2+^ on chondrocytes and osteoblasts are used to promote the integrated repair of osteochondral defects. We can also take advantage of the anti-inflammatory effects of Mg^2+^ for the treatment of osteoarthritis osteochondral defects. Through the release of a specific concentration of Mg^2+^ from the scaffold, it can not only control inflammation but also promote the regeneration of osteochondral.

## 4. Materials and Methods

### 4.1. Chemicals

Chemicals and materials (and their purity and manufacture) in the experiment are listed as follows: Magnesium chloride hexahydrate (99%, Aladdin, Shanghai, China), Collagenase Type II (Sigma Aldrich, St. Louis, MO, USA), Trypsin (Sigma Aldrich, St. Louis, MO, USA), Phosphate Buffer Saline (PBS, Hyclone, Cytiva, Logan, UT, USA), Penicillin–streptomycin (Hyclone, Cytiva, Logan, UT, USA), Fetal Bovine Serum (FBS, Biological Industries, Israel), Dulbecco’s modified Eagle’s Medium (DMEM) of High glucose (HG, 4500 mg/dL) (DEME-HG; Hyclone, Cytiva, Logan, UT, USA), Dulbecco’s modified Eagle’s Medium/Nutrient Mixture F12 (DEME/F12; Hyclone, Cytiva, Logan, UT, USA), Cell counting kit-8 (CCK8, Solarbio, Beijing, China), Crystal violet (Solarbio, Beijing, China), Calcein-AM/Propidium Iodide (PI) live/dead cell staining kit (Solarbio, Beijing, China), 4′,6-diamidino-2-phenylindole (DAPI, Solarbio, Beijing, China), FITC-phalloidin (Solarbio, Beijing, China), Lipopolysaccharide (LPS, Solarbio, Beijing, China), L-Ascorbic acid (Sigma Aldrich, A4403, St. Louis, MO, USA), β-Glycerophosphate disodium salt hydrate (Sigma Aldrich, G9422, St. Louis, MO, USA), Dexamethasone (Sigma Aldrich, D4902, St. Louis, MO, USA), Alkaline Phosphatase (ALP) Assay kit (Beyotime Biotechnology, Shanghai, China), BCIP/NBT Alkaline Phosphatase (ALP) Assay Chromogenic Kit (Beyotime Biotechnology, Shanghai, China), Alizarin red (Solarbio, Beijing, China), Trizol reagent (Yeason Biotech, Shanghai, China), Hifair^®^ II First Strand cDNA Synthesis Kit (Yeason Biotech, Shanghai, China), Hieff^®^ qPCR SYBR Green Master Mix (Yeason Biotech, Shanghai, China). Rabbit Collagen Type II ELISA Kit (Cusabio, Wuhan, China), Rabbit Collagen Type X ELISA Kit (MyBioSource, San Diego, CA, USA), Rabbit Collagen Type I ELISA Kit (Cusabio, Wuhan, China), Mouse Collagen Type I ELISA Kit (Cusabio, Wuhan, China).

### 4.2. Chondrocytes and MC3T3-E1 Culture

The Animal Ethics Committee of the School of Stomatology, Jilin University approved the procedures related to animals. Chondrocytes were isolated and cultured according to a previous study [59]. Briefly, chondrocytes were taken from the articular cartilage of the knee joints of New Zealand white rabbits of two weeks old. Firstly, the articular cartilage of the femur condyle was dissected and cut into small pieces with an approximate volume of 1 mm^3^ under aseptic conditions. The chondrocytes were separated from the cartilage tissue with the assistance of II-type collagenase. The obtained chondrocytes were cultured in DMEM/F12 supplemented with 10% FBS, 100 units/mL penicillin, and 100 mg/mL streptomycin in a cell incubator filled with 5% of CO_2_. The culture medium was updated every 2 days. The second passage of chondrocytes was used for the following experiments.

MC3T3-E1 subclone 4 was purchased from the American Type Culture Collection (ATCC) company, and cultured in DMEM-high glucose supplemented with 10% FBS, 100 units/mL penicillin, and 100 mg/mL streptomycin in a cell incubator filled with 5% of CO_2_. The culture medium was refreshed every 2 days.

### 4.3. Effects of Mg^2+^ Concentration on the Chondrocytes

#### 4.3.1. Cell Proliferation

The effect of Mg^2+^ concentration on the proliferation of chondrocytes was assessed by the CCK-8 method. Chondrocytes (1.5 × 10^3^ cells/well**)** were inoculated into each well of the 96-well plate and cultured for 24 h until updated with the 100 μL of Mg^2+^-loaded DMEM/F12 culture medium with different Mg^2+^ concentrations (1.25 mM, 2.5 mM, 5.0 mM, 7.5 mM, 10.0 mM, 12.5 mM, 15.0 mM, 20.0 mM, 25.0 mM, 30.0 mM, 60.0 mM, 90.0 mM. The stock solution of Mg^2+^ was prepared by magnesium chloride hexahydrate and stored in 4 °C). Five parallel experiments were applied for each Mg^2+^ concentration and the control group were the pure DMEM/F12 (with the concentration of Mg^2+^ being 0.7 mM). The culture medium was renewed every two days. For cell proliferation measurement, 10 μL of CCK-8 solution was added to the well and cultured for another 2 h, then the OD value was collected at 450 nm by the microplate reader at the first, third, and fifth day for each Mg^2+^ concentration and the control group of the culture medium.

The cell proliferation amounts were also observed directly by the crystal violet staining method. The as-cultured cells in the well were washed with PBS solution firstly. Then, the cells were fixed with 100 μL of paraformaldehyde for 30 min, and then re-washed with PBS solution. After that, 100 μL of crystal violet was dripped to stain the cells and re-washed with PBS solution for the image recording by a digital scanner.

#### 4.3.2. Cell Activity

The living activity of the cells was assessed via the dead-live staining method. The ultrahigh Mg^2+^ concentration groups (30 mM, 60 mM, and 90 mM) were excluded based on the CCK-8 results (due to the high inhibition effect on cell proliferation). The chondrocytes (1.5 × 10^4^ cells/well) were inoculated in 48-well plates for the Mg^2+^ concentration groups of 1.25–25.0 mM with DMEM/F12 as the control group. The culture medium was updated every 2 days. The dead and live cells were stained after being cultured for 72 h [60]. The results were recorded by a fluorescence microscope.

#### 4.3.3. Cell Morphology

Morphology of the chondrocytes was observed by a combination of FITC-phalloidin and DAPI staining method. The Mg^2+^ concentration sets and cell culture method were the same as that in Section 4.3.2. After cultured for 48 h, FITC-phalloidin and DAPI were applied to stain the F-actin and nucleus, respectively, to illustrate the morphology changes of the chondrocytes. The results were recorded by a fluorescence microscope.

#### 4.3.4. Chondrogenic-Related Gene Expressions

The effect of Mg^2+^ concentration on chondrogenic-related gene expressions (SOX9, COL II, AGC, COL X, and COL I) was evaluated by real-time quantitative PCR technique. Briefly, chondrocytes were seeded on 6-well plates with a density of 2 × 10^5^ cells/well. After cultured for 24 h, the culture media was replaced with fresh culture media containing different concentrations of Mg^2+^ (2.5 mM, 5 mM, 7.5 mM, 10 mM, 12.5 mM, 15 mM, 17.5 mM, and 20 mM). The total RNA of each group was extracted by Trizol reagent (Invitrogen, USA) after cultured for 7 days. Then reverse transcription was performed to synthesize cDNA from purified RNA using Oligo(dT) primers (Promega, San Luis Obispo, CA, USA) and SuperScript III reverse transcriptase (Invitrogen, Carlsbad, CA, USA) in accordance with the instructions of the suppliers. Finally, cDNA was subjected to real-time PCR (Applied Biosystems 7300, Foster City, CA, USA) using SYBR Green detection (PerfeCTa SYBR Green FastMix, ROX; Quanta Biosciences, Gaithersburg, MD, USA) with custom-designed primers (Takara Bio, Dalian, China; Table S1). All genes were quantified by comparison with the internal reference gene glyceraldehyde-3-phosphate dehydrogenase (GAPDH) for standardization. The 2^−ΔΔCt^ method was used to analysis of relative gene expression levels according to a reference previously published [61]. The experiment was repeated three times with three replicate wells for each sample.

#### 4.3.5. Chondrogenic-Related Protein Expressions

ELISA was performed to evaluate the protein levels of COL II, COL X, and COL I secreted by the chondrocytes cultured in varied Mg^2+^ concentrations. Briefly, chondrocytes were seeded on 6-well plates with a density of 2 × 10^5^ cells/well. After cultured for 24 h, the culture media was replaced with fresh culture media containing different concentrations of Mg^2+^ (2.5 mM, 5 mM, 7.5 mM, 10 mM, 12.5 mM, 15 mM, 17.5 mM, and 20 mM). After cultured for 7 days and 14 days, the supernatant was collected and centrifuged at 3000 rpm for 10 min at 4 °C, then stored at −80 °C for use. ELISA was performed according to the manufacturer’s protocol. The absorbance of each sample was detected using a microplate reader at the wavelength of 450 nm. Protein concentrations were calculated using a standard curve according to the instructions.

#### 4.3.6. Inflammatory-Related Gene Expressions

The effect of Mg^2+^ concentration on inflammatory-related gene expressions (IL-1β, MMP13, ADAMTS5, TIMP3, and HIF-1α) of chondrocytes was also evaluated by real-time quantitative PCR technique. We built the chondrocytes inflammatory models by the lipopolysaccharide (LPS)-induced method. Briefly, chondrocytes were seeded on 6-well plates with a density of 2 × 10^5^ cells/well. After cultured for 24 h, the culture media was replaced with fresh culture media containing different concentrations of Mg^2+^ (5 mM, 7.5 mM, 10 mM, 12.5 mM, 15 mM, 17.5 mM) and 10 μg/mL of LPS. The real-time quantitative PCR technique was used after being cultured for 24 h. The specific experimental process was the same as that in Section 4.3.4.

### 4.4. Effects of Mg^2+^ Concentration on the MC3T3-E1 Cells

#### 4.4.1. Cell Proliferation

MC3T3-E1 cells (1.5×10^3^) were seeded in 96-well plates with 100 μL of high-glucose DMEM medium (with the concentration of Mg^2+^ being 0.8 mM). Every Mg^2+^ concentration (2.5 mM, 5.0 mM, 7.5 mM, 10.0 mM, 15.0 mM, 20.0 mM, 30.0 mM, 60.0 mM, and 90.0 mM) was repeated five parallel experiments. The proliferation of MC3T3-E1 cells was assessed by the CCK-8 method and the OD values were recorded for the first, third, fifth, and seventh day, respectively. The specific experimental process was the same as that in Section 4.3.1.

#### 4.4.2. Cell Activity

The effect of Mg^2+^ concentrations on MC3T3-E1 activity was assessed by the live/dead staining method as described in Section 4.3.2. Ultrahigh concentrations of Mg^2+^ groups (60.0 mM and 90.0 mM) were excluded.

#### 4.4.3. Cell Morphology

The effect of Mg^2+^ concentration on MC3T3-E1 morphology was assessed via the FITC-phalloidin and DAPI staining method as that in Section 4.3.3. Ultrahigh concentrations of Mg^2+^ groups (60.0 mM and 90.0 mM) were excluded.

#### 4.4.4. Osteogenic-Related Gene Expressions

Osteogenic-related gene expressions of collagen type-I, RUNX2, ALP, OCN, and OPN were measured by real-time quantitative PCR technique. Briefly, MC3T3-E1 cells were seeded on 6-well plates with a density of 2 × 10^5^ cells/well. After cultured for 24 h, the culture media was replaced with osteogenic induction medium (supplemented with 50 μg/mL of ascorbic acid, 5 mM of beta-sodium glycerophosphate, and 10 nM of dexamethasone) containing different concentrations of Mg^2+^ (2.5 mM, 5 mM, 7.5 mM, 10 mM, 12.5 mM, 15 mM, 17.5 mM, 20 mM). The real-time quantitative PCR technique was used as described in Section 4.3.4 after being cultured for 7 days.

#### 4.4.5. The Protein Expressions of COL Ⅰ

The protein amounts of COL Ⅰ of MC3T3-E1 cultured in varied Mg^2+^ concentrations for 7 days were measured by ELISA. Briefly, MC3T3-E1 cells were seeded on 6-well plates with a density of 2 × 10^5^ cells/well. After cultured for 24 h, the culture media was replaced with osteogenic induction medium containing different concentrations of Mg^2+^ (2.5 mM, 5 mM, 7.5 mM, 10 mM, 12.5 mM, 15 mM, 17.5 mM, and 20 mM). After cultured for 7 days, the supernatant was collected and centrifuged at 3000 rpm for 10 min at 4 °C, then stored at −80 °C for use. The specific experimental process of ELISA was the same as that in Section 4.3.5.

#### 4.4.6. ALP Activity

The effect of Mg^2+^ concentration on the ALP activity of MC3T3-E1 was assessed by the quantitative Alkaline Phosphatase Assay kit and BCIP/NBT ALP Chromogenic Kit. MC3T3-E1 cells were seeded on 12-well plates with a density of 1.5 × 10^4^ cells/well. After cultured for 24 h, the culture media was replaced with osteogenic induction medium containing different concentrations of Mg^2+^ (2.5 mM, 5 mM, 7.5 mM, 10 mM, 12.5 mM, 15 mM, 17.5 mM, 20 mM). The high-glucose DMEM group was also cultured as the control group. The culture medium was updated every two days and the ALP activity was measured according to commercial kit instructions at the seventh day.

#### 4.4.7. Calcium Nodules Formation

The effect of Mg^2+^ concentration on calcium nodule formation of MC3T3-E1 was assessed by the alizarin red staining method. Briefly, MC3T3-E1 cells were seeded on 6-well plates with a density of 2 × 10^5^ cells/well. After cultured for 24 h, the culture media was replaced with osteogenic induction medium containing different concentrations of Mg^2+^ (2.5 mM, 5 mM, 7.5 mM, 10 mM, 12.5 mM, 15 mM, 17.5 mM, 20 mM). After being cultured for 21 days, the alizarin red staining method was used to detect the formation of calcium nodules.

### 4.5. Statistical Analysis

All the quantitative data were presented as mean ± standard deviation. Statistical Product and Service Solutions 23.0 software (SPSS, Chicago, IL, USA) was used to calculate the statistical analysis. Statistical differences of experimental results were analyzed using one-way analysis of variance (ANOVA) with Tukey’s HSD post-hoc test. Differences were considered as statistically significant at *p* < 0.05. The data were indicated with (*) for *p* < 0.05, (**) for *p* < 0.01, and (***) for *p* < 0.001, respectively. All the experiments were repeated at least three times with five replicate wells (CCK-8) of three replicate wells (PCR, ELISA, and ALP activity) for each group.

## 5. Conclusions

In summary, we have provided a systematic assessment of the effect of Mg^2+^ concentration on the cell proliferation and gene and protein expression of chondrocytes and osteoblasts simultaneously. The chondrocytes extracted from New Zealand rabbit could be promoted proliferation in the additional 1.25–10 mM Mg^2+^ supplied culture medium, while MC3T3-E1 cells could be promoted proliferation at the Mg^2+^ supplied concentration range of 2.5–30 mM. These results indicate that although chondroctytes need a lower concentration of Mg^2+^, it is still deficient in the physiological condition, especially for the osteochondral site where no blood supply provides additional Mg^2+^ for the promoting of chondral repair physiologically, while although the proliferation of osteoblast need more of Mg^2+^, it could be recruited from the neighbour sites within the bone tissues that harbour the most abundant amount of Mg^2+^ in the body. For the chondrocytes, the provided additional Mg^2+^ promotes the gene expression of SOX9 (in the Mg^2+^ concentration range of 12.5–17.5mM), Col II (12.5–20 mM), AGC (12.5–20 mM), while it also shows a tendency to promote the expression of Col X and Col I, especially at the concentration of 10 mM of Mg^2+^. The addition of Mg2+ also show a bonus effect on the inhibitory of inflame in the concentration ranges 5–17.5 mM, 5–7.5 mM, and 7.5 mM for IL-1β, MMP13, and ADAMTS5 factors, respectively. For the osteoblasts, the osteogenesis genes of ALP, Collagen I, and OCN were up-regulated in the range of Mg^2+^ concentration in 2.5–10 mM, 5–12.5 mM, and 5–12.5 mM, respectively. The ALP staining and calcium nodules assessment results also demonstrate the providing suitable concentration of Mg^2+^ could promote the osteogenesis. Although the osteochondral microenvironment is complex in the physiological condition for different joints in different animals and the Mg^2+^ needing is orchestrating by the exquisite design and functioning of the Nature, understanding the fundamental effect of Mg^2+^ on the cartilage and bone could definitely provide essential guidelines for the develop new clinical bioactive materials and medical therapeutics that benefits the patients suffering osteochondral diseases.

## Data Availability

Not applicable.

## Supplementary Materials

The following supporting information can be downloaded at: https://www.mdpi.com/article/10.3390/biomimetics7040227/s1. Figure S1: The morphology of passage one chondrocytes (A) and MC3T3-E1 subclone 4 (B); Figure S2. Chondrocytes activity after cultured in varied Mg^2+^ concentration for 72h by the Live/Dead staining (A), chondrocytes morphology after cultured in varied Mg^2+^ concentration for 24h by the FITC-phalloidin and DAPI fluorescence staining (B), the number of dead cells counted from Live/Dead staining (C), scale bar 200 μm; Figure S3. MC3T3-E1 activity after cultured in varied Mg^2+^ concentration for 72h by the Live/Dead staining (A), MC3T3-E1 morphology after cultured in varied Mg^2+^ concentration for 24h by the FITC-phalloidin and DAPI fluorescence staining (B), the number of dead cells counted from Live/Dead staining (C), scale bar 200 μm; Table S1: Forward (F) and reverse (R) primers used for quantitative RT-PCR.
