# Supplementary material for: The Biomimetics of Mg2+-Concentration-Resolved Microenvironment for Bone and Cartilage Repairing Materials Design
Source: Biomimetics (Basel). 2022 Dec 5;7(4):227. doi: 10.3390/biomimetics7040227 (PMC9775637; doi:10.3390/biomimetics7040227)
Supplement: Supplementary file 1 [file biomimetics-07-00227-s001.zip › biomimetics-2001742-supplementary.pdf]

Biomimetics of Mg^{2+}-Concentration-Resolved Microenvironment for Bone and Cartilage Repairing Materials Design

Supplementary materials

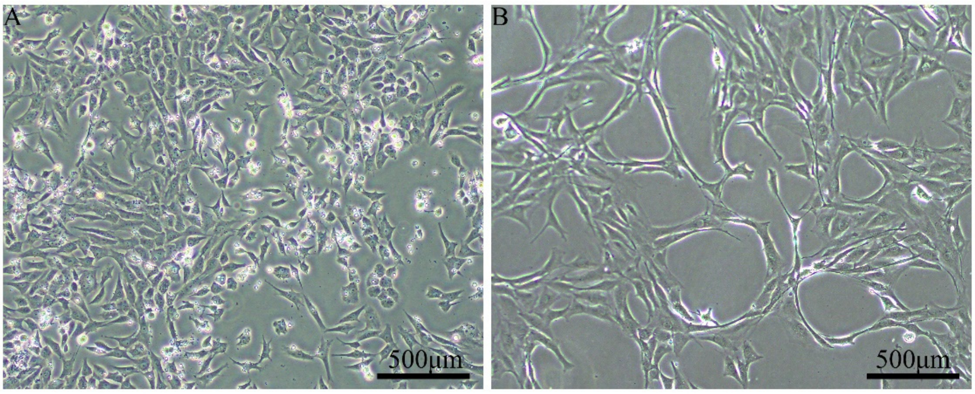

Figure S1. The morphology of passage one chondrocytes (A) and MC3T3-E1 subclone 4 (B).

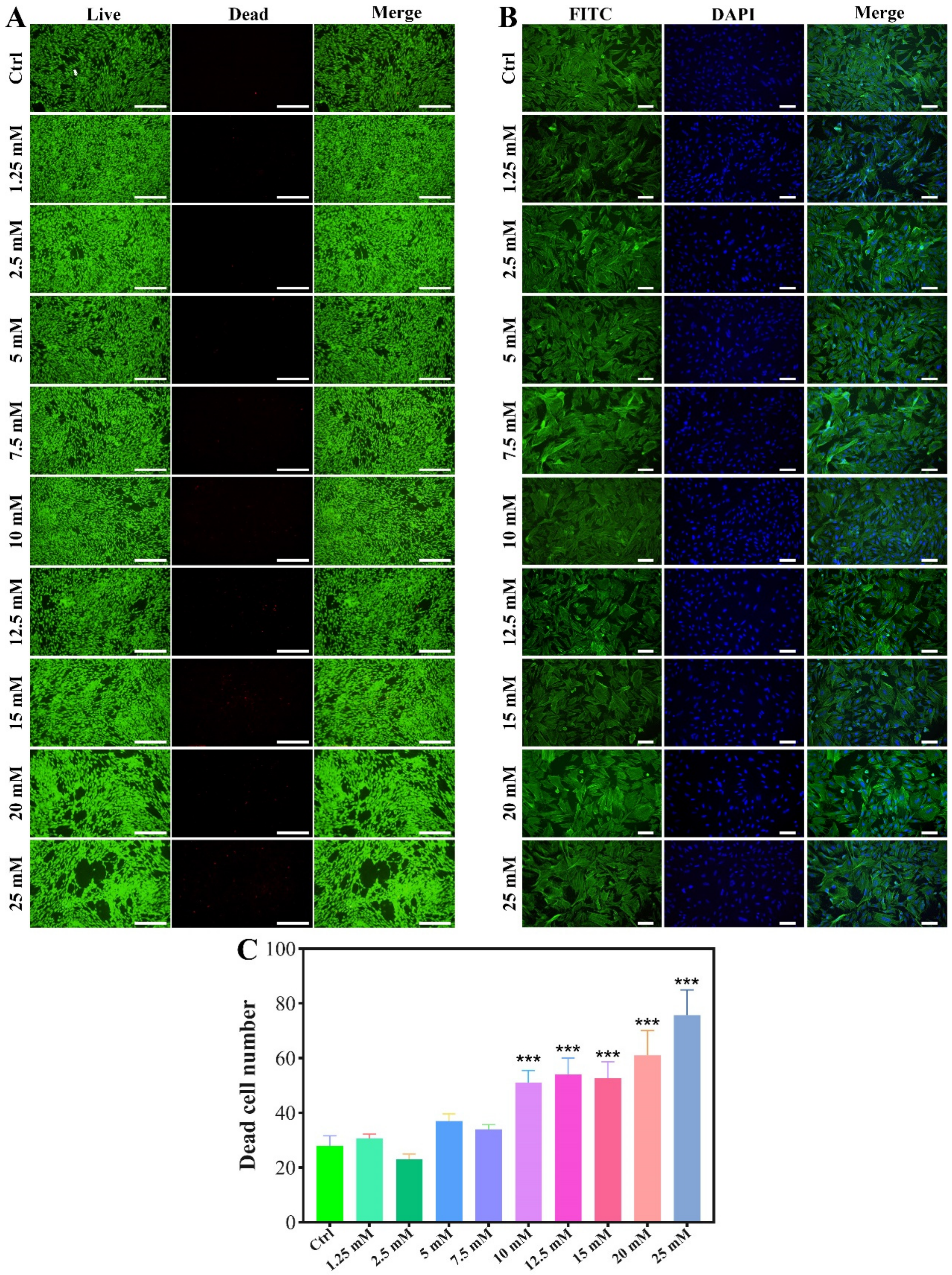

Figure S2. Chondrocytes activity after cultured in varied Mg^{2+} concentration for 72h by the Live/Dead staining (A), chondrocytes morphology after cultured in varied Mg^{2+} concentration for 24h by the FITC-phalloidin and DAPI fluorescence staining (B), the number of dead cells counted from Live/Dead staining (C), scale bar 200 μm.

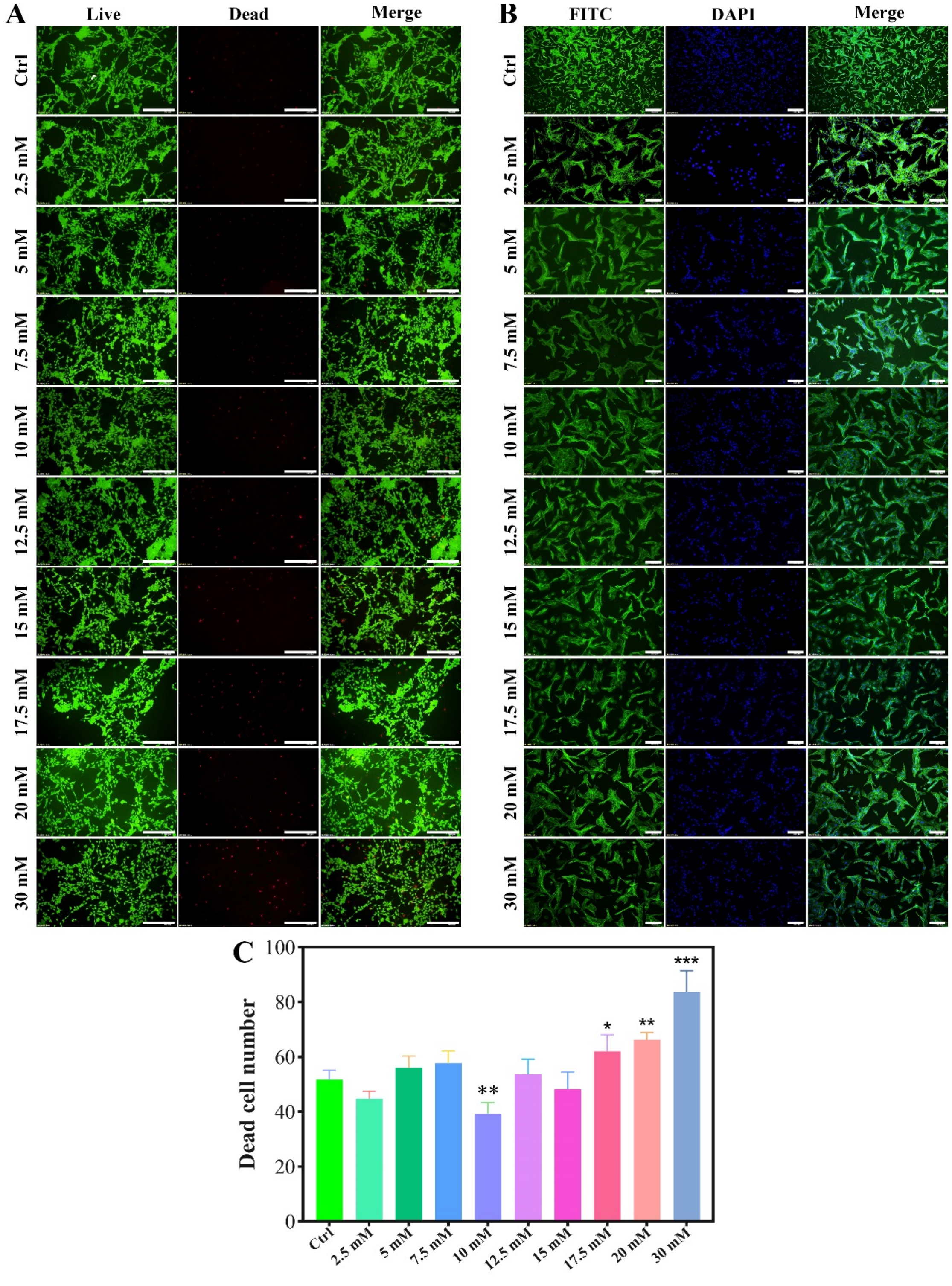

Figure S3. MC3T3-E1 activity after cultured in varied Mg^{2+} concentration for 72h by the Live/Dead staining (A), MC3T3-E1 morphology after cultured in varied Mg^{2+} concentration for 24h by the FITC-phalloidin and DAPI fluorescence staining (B), the number of dead cells counted from Live/Dead staining (C), scale bar 200 μm.

Table S1. Forward (F) and reverse (R) primers used for quantitative RT-PCR.

Gene	Primer sequence	Product length	GenBank no.
"Glyceraldehyde-3-phosphate dehydrogenase (GAPDH)"	F: cgggaaggccatcaccatct<br>R: gctccgcccttcaaatgagc	138	NM_001082253.1
Collagen type I	F: cgtggttaccctggcaatgc<br>R: agcaccgacgggaccaatag	135	NM_001195668.1
Collagen type II	F: ccatcaatggcggcttccac<br>R: gtaggccacgctgttcttgc	143	NM_001195671.1
Collagen type Ⅹ	F: ctgcaaggagagccagggtt<br>R: ggtagaccagctggcccaat	137	XM_002714724.3
Aggrecan	F: tgacccacgtcccaaagctt<br>R: cagatgtctcgccgtgggat	135	XM_008251722.2
SOX-9	F: gcggaggaagtcggtgaaga<br>R: ctgcagcgccttgaagatgg	94	XM_008271763.2
IL-1 β	F: ggcaggtcttgtcagtcgttgtg<br>R: gcagaggacgggttcttcttcaaag	131	NM_001082201.1
MMP13	F: tgcgggaatcctgaagaagaatgc<br>R: tcaagtttgcctgtcacctctaagc	96	NM_001082037.1
ADAMTS5	F: agtgtggagtatgcggaggagac<br>R: gctttgaactgtcgaaccttgatgtg	136	XM_002716775.3
TIMP3	F: gacatgctctccaacttcggctac<br>R: ggtggcgttgatggtcgtcttg	129	NM_001195682.1
HIF-1α	F: tctccattacctgcctctgaatctcc<br>R: acttgctggctgatcttgaatctgg	150	NM_001082782.1
GAPDH	F: ggttgtctcctgcgacttca<br>R: tggtccagggtttcttactcc	183	NM_008084.3
Runx2	F: gatgatgacactgccacctctgac<br>R: tgagggatgaaatgcttgggaactg	127	NM_001146038.2
ALP	F: cggcgtccatgagcagaactac<br>R: caggcacagtggtcaaggttgg	81	NM_007431.3
Collagen type I	F: gacaggcgaacaaggtgacagag<br>R: caggagaaccaggagaaccaggag	86	NM_007742.4
Osteocalcin	F: caagcaggagggcaataaggtagtg<br>R: catactggtctgatagctcgtcacaag	137	NM_007541.3
